# Stress and catecholamines modulate the bone marrow microenvironment to promote tumorigenesis

**DOI:** 10.15698/cst2019.07.192

**Published:** 2019-06-04

**Authors:** Pauline Hanns, Anna M. Paczulla, Michael Medinger, Martina Konantz, Claudia Lengerke

**Affiliations:** 1 Department of Biomedicine, University Hospital and University of Basel, Basel, Switzerland.; 2 Division of Clinical Hematology, University Hospital Basel, Basel, Switzerland.

**Keywords:** bone marrow, cancer, metastasis, leukemia, catecholamines, CXCL12/CXCR4, angiogenesis, stress

## Abstract

High vascularization and locally secreted factors make the bone marrow (BM) microenvironment particularly hospitable for tumor cells and bones to a preferred metastatic site for disseminated cancer cells of different origins. Cancer cell homing and proliferation in the BM are amongst other regulated by complex interactions with BM niche cells (e.g. osteoblasts, endothelial cells and mesenchymal stromal cells (MSCs)), resident hematopoietic stem and progenitor cells (HSPCs) and pro-angiogenic cytokines leading to enhanced BM microvessel densities during malignant progression. Stress and catecholamine neurotransmitters released in response to activation of the sympathetic nervous system (SNS) reportedly modulate various BM cells and may thereby influence cancer progression. Here we review the role of catecholamines during tumorigenesis with particular focus on pro-tumorigenic effects mediated by the BM niche.

## STRESS AND CATECHOLAMINE SIGNALING

Stress is defined as the relationship between a person and her/his environment when latter is perceived as endangering to her/his well-being. While the stimulus-based definition understands stress as the sum of effects that emerge after exerting acute or a chronic external discomfort on a subject, the response-based definition proposes stress as part of the physiological alert reaction activated by the body to better master a dangerous situation [1]. On the physiological and biochemical levels, stress involves the sympathetic nervous system (SNS) and the release of catecholamine neurotransmitters (epinephrine (EPI), norepinephrine (NOR), dopamine (DA)) from SNS fibers. Catecholamine release may differ in acute compared to chronic stress: whereas acute and chronic stress both induce EPI as well as NOR [2–5], brain concentrations of DA were instead found elevated in acute but however reduced in chronic stress [2, 6, 7]. The downstream adrenergic signaling responses activated by stress are furthermore dependent on the expression of specific adrenergic receptors (ARs) on various organs and tissues participating in the alert reaction [8, 9]. ARs belong to the class of G protein-coupled receptors and are subdivided into α- and β-ARs. Activation of α1-ARs (comprising α1a, α1b, α1d-ARs) increases intracellular calcium levels and induces vasoconstriction, while α2-AR (comprising α2a, α2b, α2c-ARs) activation inhibits intracellular cyclic adenosine monophosphate (cAMP), insulin, acetylcholine and NOR release [10]. Stimulation of β-ARs (comprising β1, β2, β3-ARs) elevates cytosolic cAMP levels and activates protein kinase A (PKA) leading to smooth muscle relaxation and lipolysis [11–13]. While β3-ARs bind EPI and NOR with the same affinity, α2 and β2-ARs are more potently stimulated by EPI and α1 and β1-ARs by NOR [14–16]. ARs can be pharmacologically modulated by drugs with selectivity for certain receptors, e.g. “selective” blockers targeting α1-ARs (e.g. prazosin), α2-ARs, (e.g. yohimbine), β1-ARs (e.g. acebutolol) or by so-called “unselective” blockers targeting for example all types of α-ARs (e.g. phenoxybenzamine), or all types of β-ARs (e.g. propranolol). AR-blockers are in routine clinical use especially for the treatment of patients with hypertension and cardiovascular disorders.

Interestingly, cancer cells may also express ARs, and enhanced levels of EPI and NOR were detected in fluids and tissues of patients with cancer [17–21], suggesting that catecholamines and adrenergic signaling are involved in cancer pathogenesis.

## EPIDEMIOLOGIC STUDIES

The relationships between stress, catecholamine levels, AR-blocker use and cancer incidence or outcome were investigated by several epidemiological studies with in part controversial results. Enhanced perceived stress (measured by self-assessment via a questionnaire) was identified as a risk factor for rectal cancer in a prospective study involving 61,563 Japanese men and women followed-up for 21 years [22]. Furthermore, elevated intra-tumor NOR levels determined by high performance liquid chromatography were associated with advanced stage and high-grade histology in ovarian carcinoma [23]. Consistently, retrospective studies indicated treatment with β-blocker to associate with increased overall and progression-free survival in patients with prostate [24, 25], breast [26–31], ovarian cancer [32] or melanoma [33, 34], and treatment with propranolol to reduce the incidence of head and neck, esophagus, stomach, colon, and prostate carcinoma in an analysis of 24,000 people followed-up for twelve years [35]. Finally, a meta-analysis of 20,898 patients with cancer (including patients from twelve studies published between 1993 and 2013, among which the studies mentioned above) indicated that β-blocker usage may associate with prolonged survival in early stage cancer patients undergoing surgical resection [36].

In contrast, other reports – also involving patients with a variety of different tumors (e.g. breast cancer [37], renal carcinoma [38], acute myeloid leukemia (AML) [39], melanoma [40–41] as well as a retrospective meta-analysis with more than 88,000 cancer patients from approximately thirty studies failed to reproduce these associations [42]. We hypothesize that these controversial results may derive from the heterogeneity among the analyzed patient groups and tumor types, stages and therapies, as well as the differences in employed β-blockers. Different types of stress may induce different catecholamine compositions (e.g. acute or chronic stress, versus exercise-induced, see also below) that influence results. Selected tumor subtypes or phases during tumorigenesis might be more or less responsive to pro-tumorigenic effects mediated by catecholamines and large meta-analyses may not sufficiently capture such effects. Prospective randomized trials focusing on defined patient subgroups, disease stages and medications are required to obtain conclusive results. Epidemiologic studies investigating β-blocker treatments in patients with cancer were recently comprehensively reviewed by Yap *et al.* [43].

## STRESS AND CATECHOLAMINES PROMOTE TUMORIGENESIS VIA CELL AUTONOMOUS AND NON-AUTONOMOUS MECHANISMS

Experimental data indicate that stress and catecholamines promote tumor growth and metastasis via both cell autonomous and non-autonomous mechanisms [17, 44–56] (see also the recent review by Qiao *et al.* [57]). In an ovarian cancer animal model, restraint stress enhanced NOR and EPI levels and thereby promoted malignant cell growth by suppressing anoikis and enhancing phosphorylation of focal adhesion kinases (FAK) [58]. Elevated housing temperature enhanced NOR levels in an orthotopic pancreatic carcinoma model thereby up-regulating the expression of anti-apoptotic B-cell lymphoma 2 (BCL-2), B-cell lymphoma-extra large (BCL-xL) and induced myeloid leukemia cell differentiation (MCL) proteins, suppressing the pro-apoptotic Bcl-2-associated death promoter (BAD) protein and inducing apoptosis resistance [59]. Similarly, in a prostate cancer xenograft model, behavioral stress increased EPI levels, induced β2-AR signaling activation and accelerated tumor progression by enhancing anti-apoptotic responses in tumor cells [60]. Finally, recent work has pointed out that catecholamines induce cytoskeleton alterations and expression of genes mediating invasive properties thereby enhancing the aggressiveness of tumor cells [45]. Molecularly, β-AR activation by catecholamines activated cAMP and downstream PKA signaling, resulting in higher Ca^2+^ efflux from the endoplasmic reticulum and finally modulation of cadherins and actin [45].

Consistent with these data, expression of different ARs has been documented on different cancer cell types and linked to cancer progression (β1: [61, 62], β2 : [17, 61, 62], β3 : [61, 63–66], and targeting of catecholamine signaling by treatment with specific β-AR inhibitors has been proposed as a potential therapeutic approach for cancer [61, 67]. In fact, treatment with specific β3-AR antagonists was shown to reduce proliferation and activate cell death in tumor cells thereby inhibiting melanoma progression in a mouse model [65].

Non-cell autonomous catecholamine-mediated pro-tumorigenic mechanisms include effects on blood and lymphatic vessels, fibroblasts, immune cells as well as different subtypes of bone marrow (BM) cells and are thus even more complex. For example, daily restraint stress was shown to activate cancer-associated fibroblasts to produce extracellular matrix components favoring ovarian cancer growth [68]. Chronic psychological stress (induced by different types of stressors) furthermore facilitated breast cancer cell metastasis to the lungs by modulating macrophage responses and the pre-metastatic niche [56]. Additionally, chronic restraint stress promoted angio- and lymphangiogenesis [49, 69] and the reorganisation of lymphatic networks within and around the primary tumor via induction of tumor-derived vascular endothelial growth factor C (VEGF-C), which in turn was found to depend on cyclooxygenase-2 (COX-2) mediated inflammatory signaling from macrophages [69]. Furthermore, in a prostate cancer mouse model NOR release in the stroma was shown to activate an angiogenic switch fueling tumor growth via the endothelial β-AR signaling pathway [70]. Consistently, β-adrenergic-mediated chronic restraint stress also enhanced leukemic burden in an acute lymphoblastic leukemia (ALL) mouse xenograft model. Interestingly, the pro-leukemogenic effect of catecholamines in this setting was not mediated by adrenergic signaling in leukemic cells themselves but rather by pro-leukemogenic modulation of host cells that interact with human ALL cells. The effects could potentially be mediated by SNS regulation of anti-tumor immune response (e.g. involving natural killer (NK) cell-mediated killing of leukemia cells) and of BM stromal cells, including osteoblasts that play a key role in the maintenance of healthy hematopoietic cells [71]. In response to stress, tumor cells furthermore showed increased release of pro-inflammatory prostaglandin E2 (PGE2) [72]. Further studies demonstrated that NOR induced activation of β3-ARs in both melanoma cells and cells of the tumor microenvironment enhanced the response of stromal macrophages and fibroblasts by inducing pro-inflammatory cytokine secretion and *de novo* angiogenesis in the tumor, thus sustaining tumor growth and aggressiveness [64]. Interestingly, pharmaceutical blockade of β3-AR could significantly decrease the tumor vasculature by activating apoptosis signaling pathways of endothelial cells in tumor blood vessels, thus reducing melanoma malignancy [65].

The pro-tumorigenic effects of stress summarized above were obtained in models studying the effects of catecholamine release associated with physical or psychological stress, or a combination of both. Interestingly, different results were observed when catecholamines released upon exercise were analyzed. Mice given the voluntary opportunity to run in an environmentally enriched cage also showed enhanced catecholamine levels, but these did not promote tumor growth [73]. Interestingly, suppressive effects on tumor growth were instead observed upon such exercise in different murine cancer models (e.g. of breast [74], pancreas [75], lung and melanoma [76]). There is no definitive molecular explanation for the different impact of stress- versus exercise-induced catecholamine increase on tumorgenesis. Possible explanations include (a) that different types of catecholamine are released in exercise compared to stress (e.g. higher induction of NOR and DA in exercise [77] and of EPI in stress conditions, perhaps due to the fact that exercise preferentially induces a response of the SNS, while stress primarily triggers an adrenal response [78]), (b) different catecholamine dynamics (intensive peaks upon exercise versus more constant enrichment under “chronic” stress with latter perhaps being only permissive for modeling of a pro-tumorgenic environment [79] and (c) confounding non-catecholamine related physiological and biochemical processes associated with exercise versus stress. Exercise-induced cancer protection could also be linked to the activation of the immune system. For example, high EPI levels induced by voluntary wheel running mobilized NK cells to the tumor site thereby reducing incidence and growth of melanoma, liver and lung tumors [76]. Catecholamine effects on the immune system are reviewed in detail elsewhere [57, 80–82].

## CANCER AND THE BM MICROENVIRONMENT

Bones and the BM are also preferred metastatic sites for different solid tumors. According to Suva *et al.*, the post mortem incidence of BM metastases is highest in patients with breast carcinoma (73%), followed by prostate (68%), thyroid (42%), lung and renal carcinoma and melanoma [83, 84]. Detectable bone metastases are a strong predictor of poor outcome and associate with 5-year survival rates of only 10% in patients with breast carcinoma [84]. In order to seed the BM, cancer cells first have to leave the primary tumor by extravasation into the circulation as disseminated tumor cells (DTCs). Next, they have to survive in the circulation until they reach their final destination, probably via a process similar to the well-described “homing” of healthy hematopoietic stem and progenitor cells (HSPCs). Upon arrival in the BM, DTCs adhere to components of this new microenvironment, which is considered a “fertile soil” for tumor cells because of its high vascularization and enhanced concentrations of pro-tumorigenic growth factors, cytokines and chemokines [85, 86]. Importantly, the BM microenvironment may facilitate tumor cell dormancy and confer chemotherapy resistance to tumor cells, thus enabling their long-term persistence despite treatments, and facilitating subsequent relapses and progression. As for example shown by Carlson *et al.* for breast carcinoma cells, DTCs occupy perivascular niches through integrin-mediated interactions driven by endothelial-derived von Willebrand factor and vascular cell adhesion molecule 1 (VCAM-1) and thereby receive selective protection against chemotherapies [87]. Interestingly, as shown for hematologic neoplasia, solid tumor cells also appear to actively modulate the BM microenvironment to facilitate its colonization. For example, high levels of soluble intercellular adhesion molecule 1 (ICAM-1), VCAM-1 and platelet-derived growth factor (PDGF) detectable in the BM plasma of untreated advanced breast cancer patients possibly contribute to DTCs escape out of the blood vessels into the BM [88]. When entering the bone to cause osteolytic or osteoblastic lesions, cancer cells initiate a cellular crosstalk that further supports tumor growth and invasion [89], often triggering a destructive auto-regulatory feedback loop promoting tumor growth. Taken together, these data indicate that cancer cells actively modulate the BM niche to facilitate its occupation. Whether vice versa a perturbed niche by itself can trigger malignancy is currently under heavy debate [89].

## THE BM NICHE IN HEMATOLOGIC MALIGNANCIES

Next to the healthy situation, the BM niche is best studied in hematologic malignancies involving malignant cells residing at this site. BM microenvironment changes are commonly observed in patients with hematologic diseases, and experimental models demonstrate an intensive crosstalk between malignant and healthy cells, suggesting that the BM niche plays important pathophysiological roles (reviewed in more detail in [90–93]). When compared to healthy controls, patients suffering from myelodysplastic syndromes (MDS) show higher CXCL12 (also named stromal cell-derived factor 1, SDF-1) levels and enhanced tumor necrosis factor α (TNFα), interleukin 6 (IL-6) and tumor necrosis factor β (TNF-β) expression in fibroblasts and macrophages, which likely contribute to disease pathogenesis [94]. However, it often remains unclear whether the observed microenvironment changes are cause or consequence of the disturbed hematopoiesis.

Leukemic cells are hypothesized to actively modulate the BM microenvironment to allow specific subpopulations of leukemia-initiating cells (so-called leukemic stem cells, LSCs) to colonize BM niches granting their long-term survival and perhaps protection against anti-neoplastic therapies (reviewed in more details in [95–98]). For example, in murine models of myeloproliferative neoplasia, malignant cells were shown to stimulate mesenchymal stromal cell (MSCs) to produce higher numbers of functionally altered osteoblasts, which then accumulate as inflammatory myelofibrotic cells to induce fibrosis and ultimately promote LSC persistence [99].

## BM ANGIOGENESIS

Many studies have suggested a role for angiogenesis not only in the pathogenesis of solid tumors but also in hematological malignancies [100–106]. The first link between leukemia progression and increased BM vascularization [107] was provided 1997, when an increased blood vessel content was demonstrated in the BM of ALL patients compared to healthy controls. A detailed analysis of BM sections from ALL patients showed irregular, albeit abundant, BM vasculature. Moreover, urine and peripheral blood samples from ALL patients exhibited elevated levels of proangiogenic basic fibroblast growth factor (bFGF) and vascular endothelial growth factor (VEGF), which correlated with the increase in BM angiogenesis [108]. In AML, increased levels of plasma VEGF were associated with worse outcome [109] and higher numbers of circulating blasts [110]. In patients with MDS, enhanced BM VEGF expression was detected in high compared to low risk patients and showed to predict evolution to transfusion dependence [111]. Increased BM microvessel density and VEGF expression were furthermore observed in patients with myeloproliferative neoplasms or lymphoma [102, 112], where enhanced BM angiogenic activity also associated with disease aggressiveness and worse outcome [113, 114].

Cells, cytokines and growth factors that maintain physiological angiogenesis are unbalanced in the neoplastic BM. Tumor cells themselves secrete cytokines (e.g. IL-6, granulocyte-macrophage colony-stimulating factor (GM-CSF), VEGF, bFGF) [115–117] to stimulate angiogenesis and promote tumor growth and dissemination [100]. In fact, the *VEGF* gene was first cloned from an AML cell line (HL-60) [118]. Malignant cells themselves furthermore may express cytokine receptors (e.g. VEGF receptors (VEGF-R)) [119, 120], through which they stimulate their own survival, proliferation and migration in an autocrine manner [115, 121].

## BM REGULATION BY CATECHOLAMINES

The BM naturally harbors hematopoietic stem cells (HSCs) responsible for sustaining the blood production. In order to fulfill this function over the whole life-span of an organism, HSCs balance dormancy and self-renewal activity with basal or demand-oriented proliferation and differentiation. HSCs reside in so-called BM niches, which are embedded in complex cellular networks that intensively communicate via molecular, biophysical (e.g. oxygen levels, blood pressure) and structural (e.g. extracellular matrix) signals [122–124]. Different BM niches have been described and reported to support the unique requirements of HSCs (as elegantly reviewed in [125, 126]). Osteoblasts producing among others osteopontin [127, 128] were initially considered major regulators of HSCs shown to reside in proximity of the endosteum [129–132]. More recent studies have questioned the importance of molecular signals deriving from osteoblasts for the regulation of HSC quiescence and rather pointed out roles of osteoblasts in the maintenance of more committed hematopoietic progenitors, and particularly in B-cell lymphopoiesis [95, 133–140]. More recently there is instead increasing evidence for the so-called vascular HSC niche. HSCs were shown to localize in proximity of sinusoids enriched for MSC activity [140, 141] and endothelial cells lining the BM vasculature and MSCs to secrete factors sustaining the maintenance and activation of HSCs and derived progenitors [133, 143–145]. The influence of the vascular niche on HSCs fate is nicely summarized in [126, 146].

Sympathetic nerve fibers are a further critical component of the BM niche. Already back in 1925 they were described by De Castro to enter the bone with blood vessels and branch to form rings around osteoblasts and osteocytes, as described in [147]. Next to a baseline routine secretion, these fibers release catecholamines to the BM space [148] in response to circadian rhythm oscillations with especially NOR levels peaking during night and EPI release instead less dependent on circadian oscillations [149]. As shown by Heidt *et al.*, chronic stress applied to mouse models induces a surplus release of NOR, which then reduces CXCL12 levels in the BM through activation of β3-ARs. Chronic stress as a consequence activates HSCs and increased their proliferation and differentiation, thereby causing increased output of inflammatory cells and inducing functional decline of HSCs [150]. Neural regulation of the BM as well as the interplay of the nerve system with the bone, BM and immunity has been recently reviewed in [151]. Several other cell types residing in the BM (e.g. immune cells [152], mast cells [153], HSPCs [154]) were also shown to produce catecholamines, which adds another layer of complexity to the regulation of adrenergic signaling in the BM.

Various cell types in the BM – among which niche cells as well as HSPCs themselves – are known to express ARs and respond to catecholamines as part of their baseline regulatory program or of demand reactions (**Table 1**) [155–158]. The circadian rhythm influences the release of HSPCs from the BM into circulation (with a maximum of mobilized HSPCs at five hours after light onset and another five hours after the onset of darkness), in part via catecholamine secretion. This cyclic release of HSPCs is in antiphase with the expression of the chemokine CXCL12 responsive for HSPCs homing and retention to the BM [156]. Interestingly, latter are regulated by core genes of the molecular clock through circadian NOR secretion by the SNS. Nerve fibers locally deliver these adrenergic signals to the BM where β3-ARs expressing stromal cells respond with CXCL12 downregulation [156]. Furthermore, granulocyte colony-stimulating factor (G-CSF) produced in response to systemic bacterial infections, mobilizes HSPCs by suppressing CXCL12 secretion from osteoblasts via NOR/EPI release [95, 156, 158, 159]. NOR release also reinforces the egress of HSPCs from the BM by acting on CAR (CXCL12 abundant reticular) cells expressing β3-ARs, in which exposure to NOR leads to degradation of specificity protein 1 (Sp1), a protein required for CXCL12 expression [156, 160, 161]. Consistently, low catecholamine levels associate with enhanced CXCL12 levels and enhanced homing and retention of CXCR4 expressing HSPCs in BM niches [161]. Thus, one major role of catecholamines in the BM is to regulate the HSPC pool via controlling their egress [161] (reviewed in more detail in [144,162–167]). Furthermore, adrenergic signals were associated with circadian leukocyte recruitment to the BM. Perivascular SNS fibers acting on β-ARs that are expressed on non-hematopoietic cells lead to differential circadian oscillations in the expression of adhesion cell molecules and chemokines, thus governing CXCR4-independent leukocyte recruitment to the BM [168]. The influence of catecholamines on cancer cells and the roles of such processes on the BM colonization by cancer cells are still understudied.

**TABLE 1:** Expression of ARs on the surface of different BM cell type in rodents.

BM cell type	Type of adrenergic receptors expressed									Reference
	α1a-AR	α1b-AR	α1d-AR	α2a-AR	α2b-AR	α2c-AR	β1-AR	β2-AR	β3-AR	
Adipocytes							+	+	+	[201]
Fibroblast like cells		+		+				+	+	[156]
HSPCs	+	+	+	+	+	+		+		[202]
Macrophages				+	+	+	+	+		[203]
MSCs	+	+		+		+	+	+	+	[156]
Osteoblasts	+									[156]
Osteoclasts		+	+					+		[156]
T lymphocytes	+	+		+	+	+		+		[85]

## HOW DO STRESS AND CATECHOLAMINES FACILITATE BM METASTASIS?

**Figure 1:**
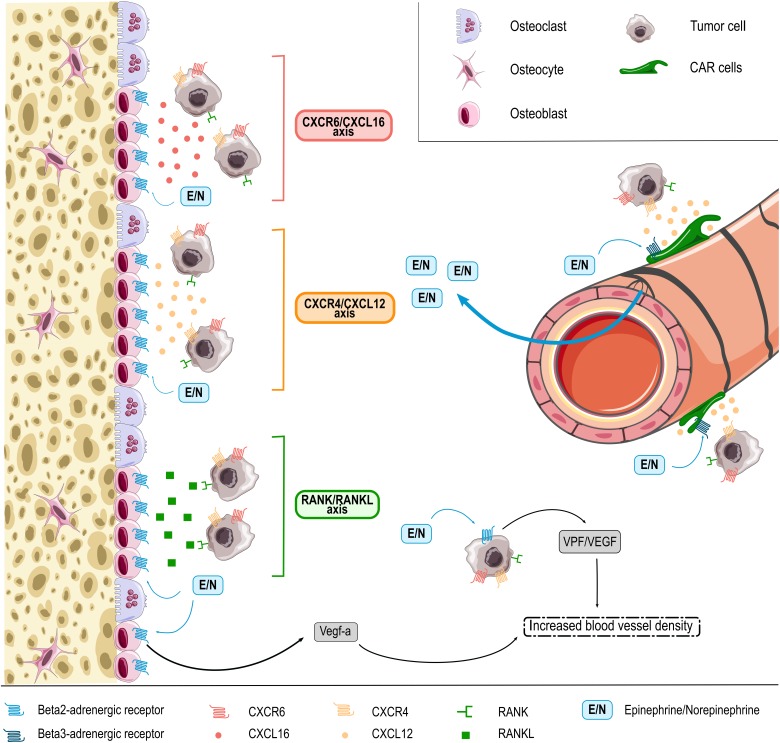
FIGURE 1. EPI and NOR (E/N, blue square) are released in the BM microenvironment from SNS fibers entering the bone with blood vessels. EPI and NOR influence interaction of tumor cells with β-ARs -expressing BM niche cells, e.g. CXCL12 abundant reticular (CAR) cells, osteoblasts and osteoclasts through different axis. In response to adrenergic signaling niche cells release (1) CXCL16 chemokine (red) that interacts with CXCR6 expressed on the surface of several tumor cells types, (2) CXCL12 (orange) that chemoattracts CXCR4 expressing cancer cells and (3) RANKL (green) protein that binds RANK-expressing malignant cells. In addition, adrenergic signaling in osteoblasts and also directly in tumor cells themselves can promote release of angiogenic factors thus promoting BM colonization by tumor cells through increased blood vessel density.

HSPC BM homing and retention is regulated by the CXCL12/CXCR4 molecular axis [169, 170]. As discussed above, this pathway is regulated by adrenergic signals, which in part are released under the influence of circadian rhythms [95, 156, 158]. While these molecular cues are best characterized for healthy HSPCs, some data suggest that they are co-used by cancer cells. As such, cancer cells may also express the CXCR4 receptor and migrate towards BM osteoblasts releasing CXCL12 (**Figure 1**) [171]. Consistently, BM areas showing metastasis also display enhanced CXCL12 expression [172]. In experimental mouse models, inhibition of CXCL12 was furthermore shown to reduce BM homing of injected multiple myeloma cells and thereby to impair disease progression [171, 172]. Another molecular axis promoting the colonization of bones by cancer cells is the receptor activator of NF-κB (RANK)/receptor activator of NF-κB ligand (RANKL) pathway [171, 173]. RANKL released by osteoblasts was shown to promote BM colonization and retention of metastatic cancer cells expressing the RANK receptor. RANK expression on tumor cells furthermore promotes their migration to the bones, while inhibition of RANKL/RANK signaling resulted in reduced bone metastasis in an experimental breast cancer model (**Figure 1**) [171, 173]. Notably, RANKL producing osteoblasts are the main source responsive to sympathetic nerves in bones because of their very high expression of β2-AR. The stimulation of these receptors by NOR was indeed shown to induce RANKL synthesis [174] and thereby promoting BM metastasis [170]. Another less prominently studied pathway is the CXCR6/CXCL16 molecular axis recently involved in homing of prostate cancer cells to the bones. While mainly expressed by antigen-presenting cells, CXCL16 is also produced by bone tissues including osteocytes and was shown to be involved in migration of CXCR6 expressing prostate cancer cells to this site (**Figure 1**) [85, 171, 175].

Cancer cells may also reach the BM without specific cues, as DTCs that are part of the circulating blood flow. Enhanced sympathetic activity in the bone microenvironment increases the density of blood vessels, which may contribute to BM colonization in patients with circulating DTCs [176]. In line, EPI and NOR were shown to increase the synthesis of pro-angiogenic factors (e.g. VEGF) thereby stimulating angiogenesis and the formation of abnormal vessels with higher permeability (**Figure 1**) (as reviewed by Chakroborty *et al.* [10]). Mulcrone *et al.* for example recently also showed that stimulation of β2-AR-expressing osteoblasts using the non-selective β-adrenergic agonist isoproterenol effectively induced VEGF-a expression thereby increasing the vascular density in the mouse BM and promoting BM metastasis (**Figure 1**) [176]. Importantly, specific blockade of the VEGF-a/VEGF-R axis abrogated the stimulatory effect of isoproterenol on tumor seeding in bones [176], suggesting direct involvement of this molecular axis in the pro-metastatic effect of catecholamines at this site.

Beyond the mechanisms discussed above, cancer cell retention in BM niche structures may be promoted by the expression of specific adhesion molecules such as cadherins, integrins or annexins that promote tumor cell binding to BM stromal cells and the bone matrix [86]. For example, osteoblasts and endothelial cells were shown to produce annexin II for which the receptor is widely expressed in cancer cells [169, 177]. Adhesion can also be mediated by E-cadherin, which was found to be expressed by cancer cells and to form adherent junctions with N-cadherin from osteogenic cells. Furthermore, αVβ3 and αVβ5 integrins expressed by tumor cells mediate binding to bone extracellular matrix proteins such as fibronectin, vitronectin or osteopontin as nicely reviewed in [171]. Interestingly, stress behavior and associated increased levels of catecholamines have been described to regulate the expression of adhesion molecules in cancer cells. For example, high levels of NOR induced a β1-integrin-mediated increase of the adhesion of human breast carcinoma cells with the vascular endothelium in an *in vitro* model of human breast cancer. Importantly, this effect was mediated by β-ARs and could be abrogated by β-blockers [178]. Furthermore, restraint stress and the associated increases in catecholamines induced increased levels of FAK in an orthotopic mouse model of human ovarian cancer, thereby affecting adhesion of tumor cells to the extracellular matrix, which contributed to cancer progression [58]. Very recently, Obradovic *et al.* showed that increase in stress hormones levels during breast cancer progression mediated activation of glucocorticoid receptors in tumor cells promoted breast cancer metastasis through induction of signaling networks and protein kinases known to facilitate breast cancer progression [179]. Whether catecholamines regulate adhesion of DTCs to the BM matrix is still under-investigated.

## COMPETITION OF NEOPLASTIC STEM CELLS WITH HEALTHY BM HSCs

As discussed above, healthy HSCs reside in specific BM niches, which however neoplastic cells modify to better serve their own requirements. As shown by imaging experiments, leukemic cells specifically engraft in microvascular BM domains showing high E-selectin and CXCL12 expression levels, where HSCs are also known to localize, indicating a possible competition between malignant and healthy cells [180]. Furthermore, transplantation of MLL-AF9 AML cells in immunodeficient mice transformed the HSC niche by reducing the density of the SNS nerve network and remodeled the BM microenvironment by depleting niche cells required for the maintenance of healthy HSCs (e.g. arteriole associated stromal cells) and expanding leukemia-supportive cells (e.g. more differentiated mesenchymal progenitors). Thus, manipulation of the adrenergic system could provide a strategy to re-install conditions favoring healthy HSCs over LSCs [181]. This notion is further supported by the work from Arranz *et al.* who showed a disturbed niche consisting in reduced numbers of sympathetic nerve fibers, supporting nestin^+^ MSCs and Schwann cells in the BM of myeloproliferative neoplasia patients as well as in mouse models. Sympathetic regulation of nestin^+^ MSCs was restored by pharmacological treatment with a β3-adrenergic agonist leading to improvement in BM fibrosis and restoration of healthy over malignant hematopoiesis [182].

Neoplastic cells from solid tumors may also outcompete healthy HSPCs from niches via selected molecular cues. Both the endosteal zone and the perivascular niche, known to harbor healthy HSPCs, are also colonization sites for tumor cells [89, 177]. This is perhaps due to the fact that neoplastic cells co-use molecular signals regulating healthy HSPCs BM homing and retention, as mentioned above. Metastatic prostate cancer cells for example were shown to use CXCR4/CXL12 [183, 184], Annexin II/Annexin II receptor [185] as well as CXCR7 [186] pathways to establish themselves in the bone [187–189]. As an example, prostate cancer cells were shown to co-localize with HSCs in the BM niche, both with a preferred binding to annexin-2 expressing osteoblasts [190]. However, prostate cancer cells showed superior ability to bind to common receptors providing them with an advantage over HSCs [191]. Interestingly, there is no direct link between the size of the primary tumor and the prevalence of DTCs in the BM of cancer patients. A limited number of available niche sites in the BM was discussed as possible cause for this phenomenon [192]. Of note, HSCs derived from animals with disseminated prostate carcinoma were found to express lower levels of niche adhesion molecules and receptors (e.g. NOTCH, angiopoietin-1 receptor (TIE2)) and transcription factors regulating HSC self-renewal and proliferation (B-cell specific Moloney murine virus integration site 1 (BMI1) and inhibitors of CDK4 A (INK4A)) [190], suggesting that these aggressive prostate carcinoma cells actively alter HSCs to vacate the niche. On the other side, as described above, DTCs take advantage of the RANKL/RANK signaling pathway induced by sympathetic activation to migrate to the BM and liberation of a few niche spaces by mechanisms described above would further give the cancer cells an advantage to settle in their new microenvironment.

## INFLUENCE ON TUMOR ASSOCIATED MACROPHAGES (TAMs) AND ANTI-TUMOR IMMUNITY

The immune system is a major player during tumor development and progression. Catecholamines, which are known to profoundly impact immune cells, may thus also exert pro-tumorigenic effects via immune modulation. For example, TAMs, which have been linked to cancer progression, metastasis and resistance to therapy [193], were shown to express ARs and respond to NOR by secretion of proangiogenic factors and matrix metalloproteinases (MMPs) promoting tumor-angiogenesis [194, 195]. Adrenergic stimulation furthermore increases cAMP and PGE2 levels within the tumor, which further mediates immune-suppressing effects [196]. More recently, EPI was shown to cooperate with inflammatory cytokines (e.g. TNFα) in the regulation of several immunosuppressive factors in both cancer cells and macrophages via COX2 activity. Consistently, EPI-dependent immune suppression was reversed by treatment with the COX2-inhibitor celecoxib [197]. Moreover, in a rat model of highly malignant syngeneic CRNK-16 induced leukemia, psychological stress or injection of stress hormones accelerated leukemia-induced death. Treatment with the β-AR blocker nadolol reversed this pro-leukemogenic effect and increased baseline survival rates. Since reduced NK cell activity was observed in animals exposed to stress hormones, impaired anti-tumor immunity was interpreted to cause the accelerated leukemia progression [198]. The influences of catecholamines on the immune system and possible links to anti-tumor immunity are elegantly reviewed elsewhere [8, 199, 200].

## CONCLUSION

Different types of stress and catecholamine release influence cancer pathogenesis [2] via regulation of the BM environment. Cancer cells of hematopoietic as well as solid tumor origin use BM niches as “safe harbor” promoting their growth and therapy resistance. They modulate the BM niche by secretion of proteins and cytokines and disrupt normal hematopoiesis by altering its adrenergic innervation. Catecholamines regulate the BM at multiple levels and further exert pro-tumorigenic effects by directly acting on AR-expressing tumor cells. Treatment with beta-blockers might be beneficial for the disease evolution in cancer patients, but the benefit is probably limited to certain tumor subtypes and stages. A better understanding of the precise molecular cues and cellular mechanisms underlying pro-tumorigenic effects of catecholamines requires further investigation but could eventually provide a rationale for more selected pharmacological intervention studies that may benefit patients with cancer.

## Abbreviations:

ALL: – acute lymphoblastic leukemia,
AML: – acute myeloid leukemia,
AR: – adrenergic receptor,
BCL-2: – B-cell lymphoma 2,
bFGF: – basic fibroblast growth factor,
BM: – bone marrow,
cAMP: – cyclic adenosine monophosphate,
COX2: – cyclooxygenase 2,
DA: – dopamine,
DTC: – disseminated tumor cells,
EPI: – epinephrine,
FAK: – focal adhesion kinase,
GM-CSF: – granulocyte-macrophage colony-stimulating factor,
HSC: – hematopoietic stem cell,
HSPC: – hematopoietic stem and progenitor cell,
IL-6: – interleukin 6,
LSC: – leukemic stem cell,
MDS: – myelodysplastic syndrome,
MSC: – mesenchymal stromal cell,
NK: – natural killer,
NOR: – norepinephrine,
PGE2: – prostaglandin E2,
PKA: – protein kinase A,
RANK: – receptor activator of NF-κB,
RANKL: – RANK ligand,
SNS: – sympathetic nervous system,
TAM: – tumor associated macrophage,
TNF: – tumor necrosis factor,
VCAM-1: – vascular cell adhesion molecule 1,
VEGF: – vascular endothelial growth factor,
VEGF-R: – VEGF receptor.
